# Transcriptional Interactions of Single B-Subgenome Chromosome with C-Subgenome in *B. oleracea-nigra* Additional Lines

**DOI:** 10.3390/plants12102029

**Published:** 2023-05-18

**Authors:** Pan Zeng, Xianhong Ge, Zaiyun Li

**Affiliations:** National Key Laboratory of Crop Genetic Improvement, National Center of Oil Crop Improvement (Wuhan), College of Plant Science and Technology, Huazhong Agricultural University, Wuhan 430070, China; uvs1951@webmail.hzau.edu.cn (P.Z.); lizaiyun@mail.hzau.edu.cn (Z.L.)

**Keywords:** *Brassica oleracea*, *Brassica nigra*, monosomic alien addition lines, aneuploidy, transcriptome

## Abstract

Serial monosomic alien addition lines (MAALs) provide an ideal system to elucidate the transcriptomic interactions between the alien chromosomes and recipient genome under aneuploidy. Herein, five available *Brassica oleracea-nigra* MAALs (CCB1, CCB4, CCB5, CCB6, CCB8), their derived *B. oleracea* plants (non-MAALs), and two parents were analyzed for their gene expressions by using high-throughput technology. Compared to parental *B. oleracea*, all MAALs showed various numbers of DEGs, but CCB8 gave much higher DEGs; the number of downregulated DEGs was slightly higher than the number of upregulated ones, except for in relation to CCB8. All derived *B. oleracea* plants also gave certain numbers of DEGs, despite these being much lower than in the respective MAALs. Compared to *B. nigra*, in all five MAALs more DEGs were downregulated than upregulated. *Trans*-effects were likely more prevailing than *cis*-effects, and these DEGs were predominantly associated with material transport by dysregulating the cellular component. Meanwhile, the orthologous genes on alien chromosomes could only play a feeble compensatory role for those gene pairs in C-subgenome, and different levels of the expressed genes had a greater tendency towards downregulation. These results revealed transcriptional aneuploidy response patterns between two genomes and suggested that *cis*- and *trans*-mechanisms synergistically regulated alien gene transcriptions after distant hybridization.

## 1. Introduction

Most angiosperms are polyploids in nature after at least one round of ancestral genome duplication and rearrangement. Studies on polyploidization indicate that this process is a momentous driving force in species evolution [1,2,3,4]. These animals usually have severely defective phenotypes, as a result of either gaining or losing individual chromosomes, resulting in low survival rates [5]. Plants (especially angiosperms) have been demonstrated to show greater tolerance than animals for interspecific hybridizations. Hence, flowering plants are applicative models to elucidate what changes relating to genome structure, relationships and functional interplay have happened in polyploid formation [6,7]. Plant interspecific crosses may eliminate one parental genome or even some their own chromosomes, resulting in the extraction of the constituent subgenomes of the natural allopolyploids or different types of aneuploid, such as nullisomic and monosomic alien addition lines (MAALs) [8,9,10,11].

Along with the rapid development of high-speed sequencing technologies, gene expression regulations and mechanisms in aneuploids are understood in more depth. For intraspecific (monosomics, nullisomics) and interspecific (alien additions) aneuploids, such as in bread wheat [12] and *B. napus* [13], not only nullisomics but also MAALs disrupt the genome balance, which generally results in profound and severe effects on transcriptional regulatory mechanisms and then responses from phenotype differentiation. In view of the widespread gene expression changes observed in aneuploid primitively, it was considered that these changes were mainly influenced by the gene dosage effect [14,15,16]. Definite evidence of the gene dosage effect was shown by studies on *Arabidopsis thaliana* [17], maize [18], human Down syndrome [19], synthetic *Brassica napus* and derivatives [20], and *B. napus*-*Orychophragmus violaceus* addition lines [21]. Then *cis*-regulatory elements and *trans*-regulatory factors were revealed [22,23]. The results in maize aneuploids [24], *Drosophila* [25], allohexaploid wheat [26], and wheat–barley 7HL addition line [27] have proved that the *cis*-regulatory elements directly affected gene expression, and *trans*-regulatory factors were usually transcription factors (TFs) or reacted on to regulating *cis* elements [28,29,30].

Thus, different types of MAALs, particularly the whole set of MAALs, should be good stocks to study the genetic interplay between the recipient genome and donor chromosome for interspecific aneuploidy. In recent years, gene expressions on alien chromosome in MAALs have been less well documented. The mice model with human chromosome 21 showed the differences in transcription systems and epigenetic machinery between humans and mice [31]. Similarly, transcription in a rat carrying human chromosome 21 illustrated the dysregulation of global gene expression, leading to anomalies in growth and development [32]. Dong et al. took advantage of two oat–maize addition lines to reveal transcriptional and epigenetic adaptation of maize chromosomes in an oat genome environment [33]. The transcriptomic analysis of *B. rapa*-*oleracea* MAALs showed that *trans*-effects were more general than *cis*-effects, and that the *trans*-effect on gene expression increased with higher levels of homology between the recipient A-subgenome and additional C-subgenome chromosomes, instead of gene numbers of extra chromosomes [13].

As one of the three cultivated *Brassica* allotetraploid species, the Ethiopian mustard (*Brassica carinata*, 2n = 34, genomes BBCC) was considered to have evolved in the highlands of Ethiopia and in the adjoining portion of East Africa and the Mediterranean coast [34], as *B. nigra* (2n = 16, BB), which grows wild in this region, and a kale-like form (2n = 18, CC), which has been in cultivation since ancient times, were possible ancestors which underwent natural hybridization in the remote past. However, after their long period of independent evolution [35,36,37,38], extant types of *B. oleracea* and *B. nigra* could not completely represent the genomic components of *B. carinata*. As an alterative, the genetic interactions and changes of the ancestral genomes in *Brassica* and other allopolyploids were extensively studied in their synthetics and aneuploids at the early stage of allopolyploidization. Previously, seven *B. oleracea*-*nigra* MAALs from the possible whole set of eight MAALs were developed from the cross between *B. oleracea* var. *alboglabra* L. H. Bailey (2n = 18, CC) (Chinese kale) and *B. nigra* (L.) Koch (2n = 16, BB), resulting in the addition of individual B-subgenome chromosomes to the complete C-subgenome [39]. These MAALs set up an interesting situation: the different additional B-genome chromosome genes will work in diverse ways in the same *B. oleracea* genome environment. This novel interspecific aneuploidy provides an excellent opportunity to elucidate the transcriptomic interactions between the alien chromosomes and the recipient genome under the aneuploidy and to detect the genetic changes of C-genome induced by the additional chromosomes in the derived *B. oleracea* plants. To achieve this, we conducted RNA sequencing for these MAALs and the derived *B. oleracea* siblings, with the aim of detecting the gene expression patterns for C- and B-subgenomes and their interactions, and providing new insights into the survival and adaption of the single B-subgenome chromosome to the recipient C-subgenome.

## 2. Results

### 2.1. DEGs Groups: MAALs vs. CC; Non-MAALs vs. CC

For more accurate alignment, we reassembled the new reference genome by using the *B. oleracea* reference genome [35] and the single corresponding B-subgenome chromosome from the *B. nigra* reference genome [36]. The distant relatedness between C- and B-genomes was helpful for aligning the reads to the prepared reference genome. To investigate transcriptional regulation, we removed adapters and low-quality reads (Q < 20) after trimming, and obtained approximately 6.00 G data size including 37.33–46.92 million clean reads from each replicate, and the alignment rates were more than 85% (Supplementary Table S1).

After filtering, DEGs were determined by employing e DEseq2 package software. Compared to the C-genome, 1348–12144 DEGs were identified in five MAALs (Table 1). Interestingly, four MAALs only had thousands of DEGs, CCB5 vs. CC (1348 DEGs, 1.99%); CCB4 vs. CC (1415 DEGs, 2.12%); CCB1 vs. CC (2074 DEGs, 3.12%); and CCB6 vs. CC (2898 DEGs, 4.32%), but CCB8 gave more than ten thousand DEGs (12144 DEGs, 17.88%). Then, by analyzing the derived *B. oleracea* data, as expected, due to the loss of extra B-subgenome chromosomes, the DEGs in the non-MAALs showed multifold decreases, CCn1 vs. CC (672 DEGs, 1.12%), CCn4 vs. CC (281 DEGs, 0.47%), CCn5 vs. CC (311 DEGs, 0.52%), CCn6 vs. CC (658 DEGs, 1.09%), and CCn8 vs. CC (4826 DEGs, 8.02%). However, among the DEGs in the MAALs the number of downregulated genes was slightly higher than the number of upregulated genes, except for CCB8 (χ^2^ test, *p* < 0.01). But in the derived *B. oleracea*, upregulated genes accounted for more than 50% of genes, except for CCn5 (χ^2^ test, *p* < 0.01). These data showed that the addition of single B-subgenome chromosomes elicited a certain extent of gene expression changes in all MAALs and even in the *B. oleracea* derivatives. The fact that a much higher number of DEGs was detected in CCB8 cannot be explained by a sampling error as the data obtained from three replicates of three different plants presented little difference. Therefore, this result must be derived from its genetic nature. The details on the DEGs in all pairwise comparisons are listed in Table 1.

### 2.2. Cis- and Trans-Effects on Gene Expression: Dysregulated Genes in Single B-Subgenome Chromosome and C-Subgenome

The MAAL and non-MAAL reads were mapped to the assembled *B. oleracea* transcriptome and the *B. nigra* sequence, with a strict alignment requirement of only one base mismatch. The reads that mapped uniquely to the *B. nigra* chromosomes, but not to the *B. oleracea* transcriptome, were used for further analysis. This strategy substantially reduced the chance that *B. oleracea* transcripts were mapped mistakenly to the *B. nigra* chromosomes, efficiently differentiated *B. nigra* from *B. oleracea* transcripts, and allowed further analysis of gene expressions specifically originating from the alien *B. nigra* chromosomes (*cis*-effects). Compared to the B-genome, 540–1545 DEGs were identified in five MAALs (Table 2). All MAALs showed great consistency for different single B-subgenome chromosomes, and the number of downregulated genes was greater than the number of upregulated genes; approximately 58–72% of DEGs were down regulated, which indicated that the *cis*-effect showed more down-regulated genes in these MAALs. Additionally, these DEGs accounted for nearly 10% of genes in each B-subgenome chromosome, B1 in CCB1 (540 DEGs, 8.75%), B4 in CCB4 (593 DEGs, 9.05%), B5 in CCB5 (680 DEGs, 8.92%), and B6 in CCB6 (635 DEGs, 9.24%), except for B8 (1545 DEGs, 20.03%). CCB8 showed the most widespread *trans*-effects with 10,599 DEGs (17.60% of the total 60,210 assembled genes), followed by CCB6 (2263 DEGs, 3.76% of the 60,210 assembled genes), and CCB1 (1534 DEGs, 2.55% of the 60,210 assembled genes), whereas the remaining two MAALs (CCB5 and CCB4) exhibited lower but similar *trans*-effects (668–822 DEGs, occupying 1.11–1.37% of the 60,210 total number of assembled genes), and the number of downregulated genes was a little higher than the number of upregulated genes, except CCB8 (χ^2^ test, *p* < 0.001, Table 2).

Similarly to the B-subgenome results, the CCB8 had more DEGs for B- or C-subgenomes than the other MAALs, which suggested that single B8 chromosome exhibited greater *cis*- and *trans*-effects on gene expressions. Whether this was associated with the expressions of some phenotypic traits of *B. nigra* origin was interesting and required further study [39].

### 2.3. Trans-Effect Dysregulation Genes Associated with Alien Chromosomes, and Their Different Transcriptional Responses to Aneuploidy

Due to the distant relationship between *B. oleracea* and *B. nigra*, it was possible to distinguish the attribution of gene expression (TPM > 1) from the gene expression profiles. For a deeper understanding of the *trans*-effect regulation, the results were obtained by assigning the DEGs to their respective chromosomes. The gene expression along each chromosome of the C-genome was distinctly impacted by different additional chromosomes in MAALs, as the extents of the *trans*-effects on each of its nine chromosomes were uneven in all comparisons. Significantly, compared to other chromosomes, the C1 chromosome was highly susceptible, but the C2 and C5 chromosomes were least affected after the addition of a foreign chromosome, something which might be related to collinearity between B- and C-genomes [40]. Thus, the genes on some chromosomes were more prone to transcriptional perturbation, indicating that the identity of additional chromosomes could affect gene expression throughout the C-genome in a specific manner (Supplementary Table S2).

**Figure 1:**
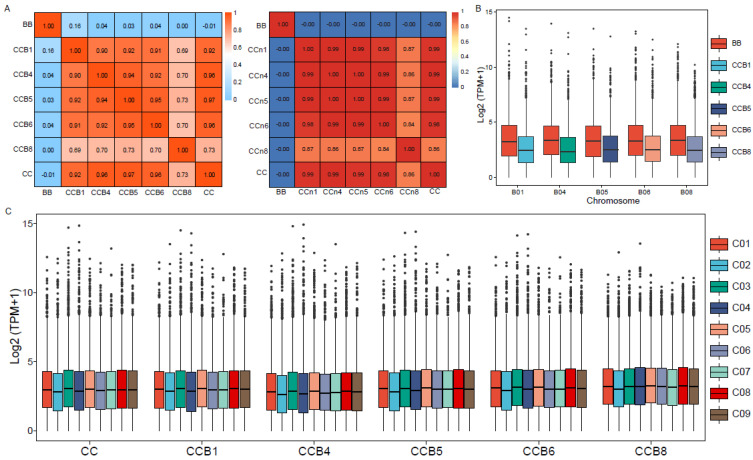
Impacts of different single B-subgenome chromosomes on gene expression patterns in the C-subgenome. (**A**) Hierarchical analysis of the global averages of all gene expression differences in the MAALs and non-MAALs. (**B**) Box plots of all expressed gene profiles (TPM > 1) on the single B- subgenome chromosome in all pairwise comparisons. The y axis represents the log2(TPM + 1). (**C**) Box plots of all expressed gene profiles (TPM > 1) on the recipient C-subgenome in all MAALs and *B. oleracea*. The y axis represents the log2(TPM + 1).

The differential impacts of different additional chromosomes on gene expression in the C-genome were also reflected by a hierarchical analysis of the similarities in terms of overall expression patterns. The differences of a global average of all gene expressions showed that CCB8 MAAL was more divergent in the overall expression patterns than the rest. Furthermore, the special MAAL was clustered together in an independent group, and the results in derived *B. oleracea* (non-MAALs) are shown in Figure 1A. Box plots of all expressed gene profiles (TPM > 1) on the donor B-subgenome, in all of the pairwise comparisons, showed that the gene expressions in the single B-subgenome chromosome were lower than that in natural *B. nigra*, confirming that the haploidy could be susceptibly disturbed in gene expression by its own change (Figure 1B). Other than for the recipient C-subgenome, due to the integrality of euploid, the box plots of all expressed gene profiles (TPM > 1) presented only subtle differences in different chromosomes (Figure 1C).

### 2.4. Functional Analysis of Differently Regulated Gene Groups

**Figure 2:**
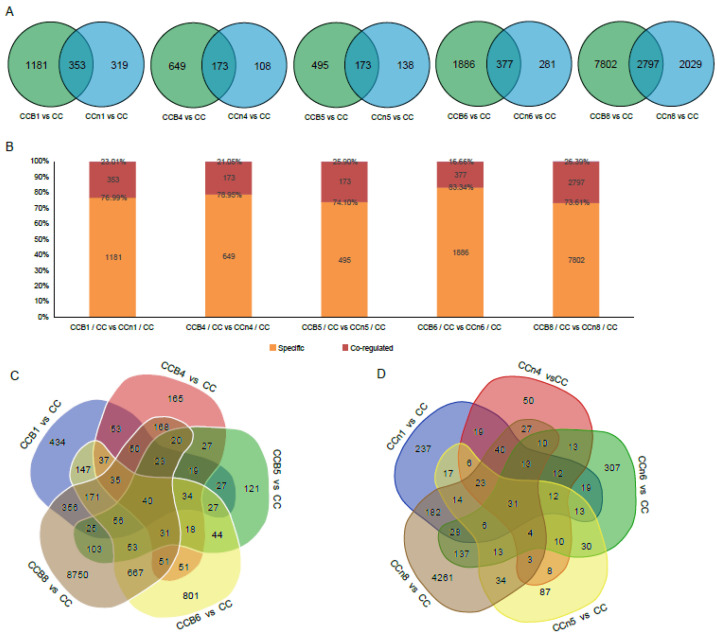
DEGs groups: MAALs vs. CC; non-MAALs vs. CC. (**A**) Venn diagrams of all DEGs in all pairwise comparisons between MAALs vs. CC and non-MAALs vs. CC. (**B**) The distributions of specific DEGs and co-regulated DEGs in all pairwise comparisons between MAALs vs. CC and non-MAALs vs. CC. (**C**) Venn diagrams of DEGs in pairwise comparisons of MAALs vs. CC. (**D**) Venn diagrams of DEGs in pairwise comparisons of non-MAALs vs. CC.

To investigate the regulatory mechanisms underlying the gene expression in alien B-subgenome chromosomes, Venn diagrams were constructed to determine whether some core genes or biological pathways of the recipient C-subgenome in MAALs responded to additional chromosomes, and the diagrams also showed the specific DEGs and co-regulated DEGs (Figure 2A). Among these pairwise comparisons, CCB8/CC vs. CCn8/CC had a significantly higher number of co-regulated DEGs (2797 DEGs, occupying 26.39% of *trans*-affected DEGs), followed by group CCB6 (377 DEGs, 16.66%), group CCB1 (353 DEGs 23.01%), group CCB4 (173 DEGs, 21.05%) and group CCB5 (173 DEGs, 25.90%) (Figure 2B). Then, 40 *trans*-affected dysregulated genes were observed in pairwise comparison between MAALs and *B. oleracea* (Figure 2C), and 31 DEGs were observed in pairwise comparison between non-MAALs and *B. oleracea* (Figure 2D). Furthermore, thirteen dysregulated genes always existed in all pairwise comparisons, including two downregulated genes in chromosomes C1, C2, and C4, and only one in chromosome C3, two upregulated genes in chromosome C3 (one of them was opposite in CCB8 and CCn8), and four in chromosome C6 (one of them was opposite in CCB8). All the results demonstrated that the additional *B. nigra* chromosomes might affect *B. oleracea* components for gene translation through an unknown mechanism, and these effects could be inherited. Although the global transcriptional aneuploidy response patterns showed general uniformity, there was no significant overlap in individual dysregulated genes between different pairwise comparisons of each MAAL vs. CC. We tried to annotate these 13 co-dysregulated genes into the public Gene Ontology (GO) database to elucidate whether these genes were involved in one or more specific biological pathway(s). Unfortunately, due to the limited number of genes used in the analysis, these genes failed to be clustered into any GO terms or KEGG pathways. However, we found the putative functions of the co-dysregulated genes (Table 3). According to their ortholog gene functions in *A. thaliana*, the downregulated DEGs were mainly involved in “magnesium ion transmembrane transporter activity”, and “nucleic acid binding translocator”, implying that the genetic function in MAALs was disturbed after the addition of the extra chromosome. In addition, the upregulated DEGs were involved in “signal transduction”, and “defense response”, which agreed with the results showing that MAALs responded to stress by increasing their levels of gene expression [13].

**Figure 3:**
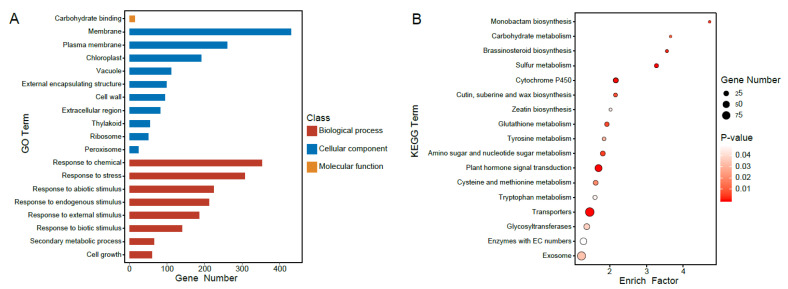
GO enrichments and KEGG analysis for co-regulated DEGs in all pairwise comparisons and DEGs identified in more than two comparisons. (**A**) A total of 19 GO terms that are particularly involved in cellular components and biological processes and are enriched with DEGs in all pairwise comparisons (FDR, *p* < 0.05). The x axis represents the gene number. (**B**) KEGG analysis of DEGs that are identified in more than two pairwise comparisons. The x axis represents enrich factor.

Then we changed the gene annotation strategy by detecting the DEGs in at least two pairwise comparisons in all pairwise comparisons to investigate the functions and to find whether different gene groups were involved in distinct or similar biological pathways. Then, 19 GO terms were enriched with these DEG groups in all comparisons (*p* < 0.05, Figure 3A) and were mainly associated with cellular components, including membrane structure, such as “membrane” (gene number: 430) and “plasma membrane” (gene number: 260), organelle, such as “chloroplast” (gene number: 191), “thylakoid” (gene number: 54), and “ribosome” (gene number: 50), and biological process, such as “response to chemical” (gene number: 353) and “response to stress” (gene number: 307). The term “carbohydrate binding” (gene number: 14) was also detected; it was the only one associated with molecular function. These results suggested that additional B- subgenome chromosomes generally had a consequence on material transport by dysregulating the cellular component, and those DEGs in all comparisons belonging to distinct groups were involved in diverse cellular pathways (*p* < 0.05, Figure 3B).

### 2.5. Expression Analysis of Orthologous Genes from MAALs in Parental Genomes

As *B. oleracea* and *B. nigra* which were diploid species of U triangle [41] were derived from the same ancestor with several paleo-polyploidy events, there were a large number of orthologous gene pairs [35,36,42]. For this reason, we were able to detect the genes on single B-subgenome chromosomes and corresponding orthologous genes in the C-subgenome for each MAAL. The chromosomes B01, B04, B05, B06, B08 had 2844, 2928, 3309, 3238, 3360 orthologous gene pairs with the C-subgenome, respectively. Owing to the limitations of sampling, we removed unexpressed genes (TPM < 1) in either genome, and the proportion of remaining dysregulated genes in CC were 4.20–29.94% (Table 4).

The single copy of the alien B-subgenome chromosome in MAAls had only a half of the genes found in the diploid state of *B. nigra*. No matter how they expressed, most of orthologous genes from the additional B-subgenome chromosome were downregulated, but only 15.52% of orthologous genes were downregulated in chromosome B08, even though they were upregulated in the C-subgenome, on account of the number orthologous genes of chromosome B08. Conversely, the number of upregulated orthologous genes in the C-subgenome was higher than the number of downregulated genes except for CCB6 and CCB8, suggesting that the gene expression of the recipient genome disturbed by the alien chromosomes could actively increase to maintain the genetic balance. As for the expression relationship between orthologous genes in two genomes, we showed that if the orthologous genes were upregulated in the C-subgenome, more than 30% of them would be downregulated in the B-subgenome, except for CCB8, but if the orthologous genes were downregulated in the C-subgenome, only about 1.01–7.75% of them would be upregulated the in B-subgenome. Additionally, these results indicated that the orthologous genes in the B-subgenome could play a certain compensatory role in orthologous gene pairs, but more of them might have an inhibiting effect on orthologous gene expression in the C-subgenome.

### 2.6. Bias to Downregulation for Different Levels of Expressed Genes and Their Maintenance

The addition of an alien chromosome could affect the transcriptional euploid response. To understand the variation and analyze the transcriptional aneuploidy process in MAALs, we detected the different expression levels of the genes and estimated whether recipient genome was or was not similarly impacted by these alien chromosomes of the B-subgenome, and, with the loss of the alien chromosome, whether the influence was maintained in the derived *B. oleracea*. Based on the gene expression level (TPM > 1) in the parental *B. oleracea* (CC), we classified the *trans*-effect dysregulated genes into three groups with low (1 < TPM < 10), medium (10 < TPM < 100), and high (TPM > 100) expression levels. More than half of the expressed genes (13,146 genes, representing 53.12% of the genes with TPM > 1) belonged to the first group, about 40% of them (10,262 genes, 41.47%) were in the medium expression level group, and relatively few genes (1340 genes, 5.41%) were in the high expression level group. Compared to CC, no matter which group they belonged to, there were more dysregulated genes were than downregulated ones (except for CCB8 vs. CC in the low group), and CCB8 (33.04–49.33%) and CCn8 (15.68–23.06%) had the largest range of variation, while CCn5 (0.82–1.05%) presented a relatively low range of variation. In MAALs, the group of low expression genes gave rise to the highest proportion of *trans*-affected DEGs (2.30–33.04% of the total number of low expression genes), indicating that the group was more susceptible to the impact of alien chromosomes. However, the other two groups did not exhibit explicit uniformity to the *trans*-affected DEGs, suggesting that the influence on the recipient genome was probably determined by foreign chromosomes (Table 5).

Likewise, we extracted the DEGs in the group MAALs vs. CC and analyzed these gene expression profiles between the derived *B. oleracea* (non-MAALs) and CC (Table 6). Obviously, only a few of them (0–3.11%) were opposite, 32.02–70.54% of the DEGs were maintained and rest of them returned to normal, illustrating that whether the alien chromosome existed or not, some of the dysregulated genes could be hereditary and the plants with the recovered euploidy could not make a full recovery immediately. This result agreed with the observation that the gene expression in plants with the exchanged genetic material would be dysregulated, and that these changes existed instantaneously, but some of them could be kept and heritable [43].

### 2.7. No Obvious Dysregulation Domains in MAALs

**Figure 4:**
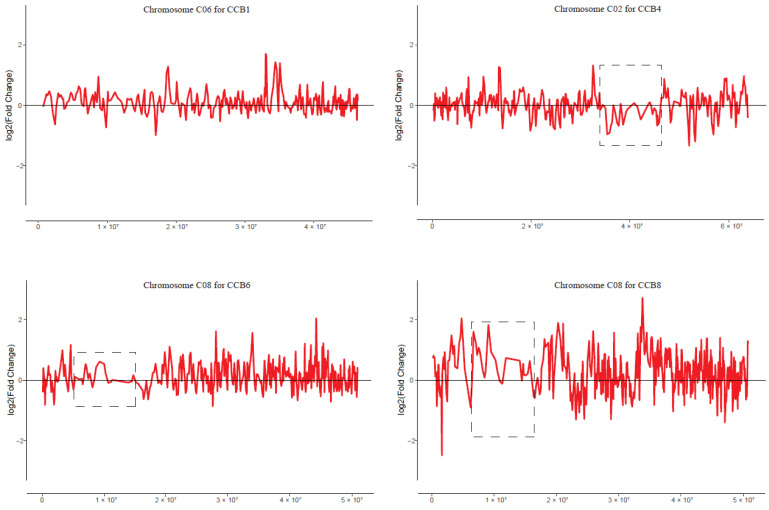
Dysregulated domains of gene expression in different chromosomes in some pairwise comparisons. Red lines denote the smoothed distribution for the differentially expressed genes in these selected chromosomes, as representatives, using the Lowess function of R. Dysregulated domains that are exhibited in dotted boxes are clearly observed in only three chromosomes in three pairwise comparisons (chromosome C02 in CCB4, chromosome C08 in CCB6, and chromosome C08 in CCB8). The y axis represents the log2(fold change) value of TPM between the MAALs and CC. The x axis represents the sorted positions of genes on these chromosomes.

The dysregulated genes might be clustered as domains on chromosomes to form gene expression dysregulation domains (GEDDs), as shown in human trisomy 21 [19] and the nullisomics in *B. napus* [44], but not in the other studies, such as *B. rapa*-*oleracea* MAALs [13] and oat–maize MAALs [33]. To detect the GEDDs in these MAALs and identify the affected chromosomes, we attempted to smooth the distributions of gene expression fold changes (log2 ratios) along each normal chromosome for each MAAL, using the Lowess function of R to detect potential GEDDs. Unfortunately, we only observed three dysregulated regions on different chromosomes (Figure 4), two of them on chromosome C08, but this GEDD was not observed in other comparisons, suggesting that there was an indeterminacy for aneuploidy and the common feature for these GEDDs might be found by more aneuploidy in different comparisons. Moreover, there was a hypothesis which needed further verification that the three-dimensional structure of alien chromosome was broken first and transmitted to other paired chromosomes.

### 2.8. Validation of DEGs by qRT-PCR Analysis

**Figure 5:**
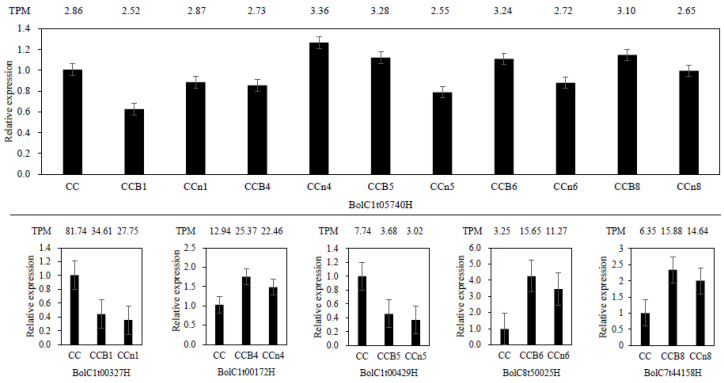
qRT-PCR confirmation of randomly selected DEGs. Expression values (transcripts per kilobase million, TPM) of 6 genes revealed by RNA-seq analysis are shown by the numbers on the top of each gene while the relative expression levels revealed by qRT-PCR are represented by the columns. Columns and bars represent the means and standard error (n = 3), respectively.

To validate the transcriptome data, we selected five DEGs that were prepared for qRT-PCR assays (Figure 5). One of them (BolC1t05740H) expressed in all materials associated with *B. oleracea* (the parent, all MAALs and all non-MAALs), the rest of them were distributed to each group (BolC1t00327H in group CCB1 and CCn1, BolC1t00172H in group CCB4 and CCn4, BolC1t00429H in group CCB5 and CCn5, BolC8t50025H in group CCB6 and CCn6, BolC7t44158H in group CCB8 and CCn8). All these results showed similar expression patterns to those determined by transcriptome. Taken together, these results were consistent with transcriptome analysis, suggesting that the transcriptome results were reliable.

## 3. Discussion

The ongoing development of high-throughput sequencing technologies allows for transcription regulation to be studied across entire genomes. Multi-omics analysis can help us to comprehensively investigate the biological processes in the study [45,46]. From the latest reports, if the genetic material of the species changes, such as by gaining or losing some chromosomes, the phenotype and growth will change considerably, and these changes exert a negative impact in most cases, even leading directly to death. However plants with high tolerance to aneuploidy can be a good model to analyze genome-wide gene expressions [12,13,31,32,33,47]. Phenotypic consequences in aneuploidy might result from the gene dosage effect [14,48] or *cis*- or *trans*-effects on global alterations of the regulatory system [49]. This means we should pay close attention to the changes in transcriptional expression and find the causes associated with gene copy number or other effects. Researchers have devoted themselves to discovering a relatively complete model to explain the gene expression in aneuploidy. Unfortunately, there was a consistent trend that MAALs exhibited prevalent *trans*-effect gene expression, compared to parents [13,33]. Similarly, our results showed this phenomenon, as the changes in transcriptional expression brought about by the different alien chromosomes were diverse. The detection of orthologous gene pairs from two parental genomes confirmed that single foreign chromosomes only played a weak role in dosage compensation for the recipient genome.

Generally, the expounded analogous cellular pathways occurred in different forms of gene expressions in aneuploidy cells and affected the stereotypical antiproliferative response as the genes associated with the cell proliferation were largely downregulated and then the genes involved in the response to stress were upregulated to survive [50]. The MAALs disrupted the normal cell cycle and cell proliferation with the addition of alien chromosomes, and had phenotypic and growth difference [39]. From different pairwise comparisons between MAALs, only 13 co-regulated genes were detected and associated with negative proliferation or enhanced stress. GO and KEGG analyses of co-regulated DEGs had similar results indicating that these cellular pathways were most related to response to stress. This might be the result of self-protection mechanisms that plants have evolved to ensure their own reproduction.

We tried to classify the DEGs according to gene expression level (Table S2), and observed that the downregulated genes were widespread in all pairwise comparisons (except CCB8 vs. CC in the low group). However, previous research showed that highly expressed genes were downregulated and lowly expressed genes were upregulated in *B. napus* nullisomics [44] and Down syndrome [32], which might be attributable to the close relationship between the A- and C-subgenomes in *B. napus*, and showed that the extra chromosome was the homologous pair in Down syndrome. However, the transcriptomic result in oat–maize addition lines was consistent with our result [33]. These different conclusions suggested that in the genomic compositions of aneuploids, the relationship between different genomes could influence the gene expressions from aneuploidy due to dosage compensation.

Some phenotypic features of trisomy 21 were observed to be related to the GEDDs or the extra chromosomes [19], but the recent study revealed that GEDDs occurred whenever gene expression changed and resulted from mammalian genome organization [51]. Such domains were also deduced in plants aneuploids, including the nullisomic *B. napus* [44]. However, in the results from trisomy 5 in *Arabidopsis thaliana* [17], hexaploid wheat aneuploidy [26], *B. rapa*-*oleracea* MAALs [13] and the present MAALs showed no evidence for such coregulated aggregated expression domains, indicating that the GEDDs might be specific to certain types of aneuploids and certain chromosome domains.

## 4. Materials and Methods

### 4.1. Plant Materials

Seven *B. oleracea*-*nigra* MAALs (2n = 19, CC + 1B 1, 3–8, without the one with B2 chromosome) were previously obtained from the cross between *B. oleracea* var. *alboglabra* L. H. Bailey (2n = 18, CC) and *B. nigra* (L.) Koch (2n = 16, BB) [39,52]. The selfed progenies of these MAALs were grown in the greenhouse in Huazhong Agriculture University (Wuhan, China). Fifty progeny plants were established for MAALs B1, B4, B5, one hundred plants for MAALs B6 and B8, and at least five additional plants were identified for each of the six MAALs. Four of the derived *B. oleracea* plants (non-MAALs) (CCn1, CCn4, CCn5, CCn6, CCn8) were randomly selected for RNA sequencing. However, only 30 plants were established for B3 and B7, and no additional plants were detected, possibly due to the low transmission rate of the additional B-subgenome chromosomes, and no further work was carried out in relation to them.

In order to screen the additional plants with alien chromosomes of the B-subgenome, the target young plants were first identified by PCR amplification of the chromosome-specific SSR (simple sequence repeats) markers that were distributed on both arms of the target chromosomes. Then, cytological analysis of chromosome counting and fluorescence in situ hybridization (FISH) with the B-genome-specific centromere probe [39,53,54] was conducted to determine the karyotype integrity of the target plants (Supplementary Figure S1). The ovaries from young flower buds were collected and treated with 2 mM 8-hydroxyquinoline for 3.5 h at ~25 ℃, and then fixed in Carnoy’s solution (3:1 ethanol: glacial acetic acid, *v*/*v*) and stored at −20 ℃ for cytological analysis. The cytological observation was conducted according to Li, et al. [55], and FISH analysis was carried out as described by Cui, et al. [52].

### 4.2. RNA Extraction and RNA-Seq

Considering the growth rate of the plants, there were minimal phenotypic differences between *B. oleracea* plants (including non-MAALs) and CCB MAALs at the six-leaf stage, and the fourth newly expanded leaves without petioles from at least three target plants were collected and immediately stored in liquid nitrogen for RNA extraction, until their chromosome complements were determined by cytological observations. Three biological replicates for each type of materials were prepared to assess gene expression. According to the manufacturer’s protocol, total RNA was extracted using a commercial RNA kit (Tiangen, Beijing, China). RNA levels and integrity were tested with agarose gel electrophoresis, a nanophotometer spectrophotometer (IMPLEN, Westlake Village, CA, USA), a Qubit2.0 fluorometer precise (Life Technologies, Carlsbad, CA, USA), and an Agilent 2100 bioanalyzer (Agilent Technologies, Santa Clara, CA, USA). The clustering of the index-coded samples was performed on a cBot Cluster Generation System using TruSeq PE Cluster Kit v3-cBot-HS (Illumia, Shanghai, China) according to the manufacturer’s instructions. After cluster generation, the library preparations were sequenced on an Illumina Novaseq platform and 150 bp paired-end reads were generated. The raw data (raw reads) in fastq format were firstly processed through in-house perl scripts. In this step, clean data (clean reads) were obtained by removing reads containing adapter, reads 1 containing ploy-N and low quality reads from the raw data. At the same time, the Q20, Q30 and GC content of the clean data was calculated. All the downstream analyses were based on high-quality clean data.

### 4.3. Transcriptome Analysis

We trimmed the paired-end reads to remove adaptor sequence and low-quality reads, and removed trimmed reads < 100 bp size by Trimmomatic (v0.39) [56]. Then the clean reads were mapped to the *B. oleracea* HDEM genome sequence (http://www.genoscope.cns.fr/plants, accessed on 10 July 2022) and to *B. nigra* (http://cruciferseq.ca, accessed on 10 July 2022) using Hisat2 (v2.1.0) [57]. StringTie (v2.1.4) [58] software was to calculate the read count and gene expression levels TPM (Transcripts Per Kilobase of exon model per Million mapped reads) of each gene. The differentially expressed genes (DEGs) were identified by the R program DEseq2 (v1.34.0) [59] package and the filtration parameters were|log2FoldChange| ≥ 1, Padj < 0.05 and average TPM of any group ≥ 1.

### 4.4. Quantitative RT–PCR

We randomly selected 6 genes for qRT-PCR assays. The total RNA of the leaves was extracted using an RNAprep pure Plant Kit (Tiangen, DP441) following the manufacturer’s instructions. RNA was treated with RNase-free DNase I to remove genomic DNA. Firststrand cDNA was synthesized using a RevertAid First Strand cDNA Synthesis Kit (Thermo Scientific, Waltham, MA, USA). Gene-specific primer sequences and detailed information are given in Table S3. qRT-PCR reactions were conducted using SYBR Green Realtime PCR Master Mix (Toyobo, Osaka, Japan) with a CFX96 Touch Real-Time PCR Detection System (Bio-Rad, Hercules, CA, USA). The thermal cycler was performed as follows: 1 cycle of 95 °C 1 min; followed by 40 cycles of 95 °C for 10 s, 60 °C for 30 s, and 72 °C for 30 s. The 2^−∆∆Ct^ method [60] was used to analyze the results with CFX Manager software (Bio-Rad, Hercules, CA, USA). Three biological replicates (with three technical replicates for each biological replicate) were analyzed for each sample.

### 4.5. The GO and KEGG Enrichment Analysis

All proteins in the *B. oleracea* genome and the *B. nigra* genome were aligned to TAIR10 (*Arabidopsis thaliana*) (https://www.arabidopsis.org/, accessed on 19 July 2022) proteins to identified homologous genes by BLASTP (v2.10.0) [61] with an E-value threshold of 10^−5^. The GO annotations of each gene in *B. oleracea* genome and *B. nigra* genome were corresponded to homologous genes in *A. thaliana* and the KEGG annotation of each gene used online KEGG database (https://www.genome.jp/kegg/, accessed on 19 July 2022). The GO and KEGG enrichment analysis used TBtools (v1.0987663) [62] software and ggplot2 (v3.3.6) package for plotting.

### 4.6. Data Statistics and Visualization

To check the statistical significance of the dysregulated expression between each MAAL and CC, a Chi-square test was used with a 0.05 *q*-value as the cutoff. To determine whether the *trans*-acting effects on gene expression were associated with the gene number of extra chromosomes, the Pearson correlation coefficient (PCC) with an FDR-adjusted *p*-value < 0.05 as the cutoff was used.

Fluorescence in situ hybridization images was captured using a computer-assisted fluorescence microscope with a CCD camera (Axio Scope A1, Zeiss, Oberkochen, Germany). Images of the gene expression analysis were generated by plotting the functions of the R-project and were then composed by Adobe Illustrator version CC.

## 5. Conclusions

As a result of its better drought tolerance and resistance to fungal diseases, *B. carinata* is gaining ground in semi-arid areas of southern Europe, western Canada, Australia and India, and also serves breeding as the germplasm [35]. In particular, these MAALs were distinguishable morphologically from each other, as they expressed the characters from *B. nigra* differently [39]. The comprehensive transcriptome analysis of *B. oleracea*-*nigra* MAALs provided novel insights into the gene expression patterns of recipient C-subgenome to the individual B-subgenome chromosomes, and provided a deep understanding of the functional interplay between two parental genomes for the evolutionary formation of the allotetraploid *B. carinata*.

## Figures and Tables

**Table 1:** Summary of up- and down-regulated genes in all pairwise comparisons between MAALs and CC, non-MAALs and CC.

Samples	Up Regulated Genes	Ratio (%)	Down Regulated Genes	Ratio (%)	Total	Ratio (%)
CCB1 vs. CC	861	41.51	1213 **	58.49	2074 ^A^	3.12
CCB4 vs. CC	547	38.66	868 **	61.34	1415 ^A^	2.12
CCB5 vs. CC	559	41.47	789 **	58.53	1348 ^A^	1.99
CCB6 vs. CC	1336	46.10	1562 **	53.90	2898 ^B^	4.32
CCB8 vs. CC	6167	50.78	5977	49.22	12144 ^C^	17.88
CCn1 vs. CC	354	52.68	318	47.32	672 ^a^	1.12
CCn4 vs. CC	165 **	58.72	116	41.28	281 ^a, b^	0.47
CCn5 vs. CC	154	49.52	157	50.48	311 ^a^	0.52
CCn6 vs. CC	424 **	64.44	234	35.56	658 ^b^	1.09
CCn8 vs. CC	2642 **	54.75	2184	45.25	4826 ^a^	8.02

** The group of up-/down- DEGs is significantly higher than oppositely regulated genes (chi-square, *p* < 0.01). ^A, B, C; a, b^ Different groups were calculated by chi-square (*p* < 0.001).

**Table 2:** *Cis*-and *trans*-affected dysregulated genes in all pairwise comparisons between MAALs and CC.

Comparisons	*Trans*-Effects			*Cis*-Effects		
	Up- (%)	Down- (%)	Total (%)	Up- (%)	Down- (%)	Total (%)
CCB1 vs. CC	694 (45.24)	840 (54.76) **	1534 (2.55) ^A^	167 (30.93)	373 (69.07) **	540 (8.75) ^A^
CCB4 vs. CC	379 (46.11)	443 (53.89) *	822 (1.37) ^A^	168 (28.33)	425 (71.67) **	593 (9.05) ^A^
CCB5 vs. CC	332 (49.70)	336 (50.30)	668 (1.11) ^A, B^	227 (33.38)	453 (66.62) **	680 (8.92) ^A^
CCB6 vs. CC	1121 (49.54)	1142 (50.46)	2263 (3.76) ^A, B^	215 (33.86)	420 (66.14) **	635 (9.24) ^A^
CCB8 vs. CC	5522 (52.10) **	5077 (47.90)	10599 (17.60) ^B^	645 (41.75)	900 (58.25) **	1545 (20.03) ^B^

^A, B^ Different groups were calculated by chi-square (*p* < 0.001). ** The group of up-/down- DEGs is significantly higher than oppositely regulated genes (χ^2^, * *p* < 0.05, ** *p* < 0.01).

**Table 3:** The putative gene functions of co-dysregulated genes in all pairwise comparisons of MAALs vs. CC and non-MAALs vs. CC.

Gene ID	Up/Down	Putative Orthologs in *A. thaliana*	Gene Functions
BolC1t04413H	Down	AT3G19640	Magnesium ion transmembrane transporter activity, metal ion transmembrane transporter activity
BolC1t05746H	Down	AT1G60720	RNA-directed DNA polymerase (reverse transcriptase)-related family protein
BolC2t09633H	Down	AT3G32904	Unknown protein
BolC2t11658H	Down	AT1G10000	Ribonuclease H activity, nucleic acid binding
BolC3t13980H	Up	AT2G32260	Phosphorylcholine cytidylyltransferase
BolC3t14199H	Up/Down (B8/n8)	AT5G53160	Regulatory components of ABA receptor 3
BolC3t15755H	Down	AT4G09340	SPla/RYanodine receptor (SPRY) domain-containing protein
BolC4t23401H	Down	AT2G31470	F-box and associated interaction domain-containing protein
BolC4t24470H	Down	AT1G63670	Protein of unknown function (DUF3741)
BolC6t37425H	Up	AT2G32260	Phosphorylcholine cytidylyltransferase
BolC6t39384H	Up	AT1G33590	Leucine-rich repeat (LRR) family protein
BolC6t39403H	Up/Down (B8)	AT2G33150	Peroxisomal 3-ketoacyl-CoA thiolase 3

**Table 4:** The expression of orthologous genes from MAALs in parental genomes (*B. oleracea* and *B. nigra*).

MAALs	Up in CC					Down in CC				
	Unexpressed in BB (%)	Down in BB (%)	Up in BB (%)	Unchanged in BB (%)	Total (%)	Unexpressed in BB (%)	Down in BB (%)	Up in BB (%)	Unchanged in BB (%)	Total (%)
CCB1	13 (22.42)	24 (41.38)	6 (10.34)	15 (25.86)	58 (2.04)	16 (20.25)	36 (45.57)	4 (5.06)	23 (29.12)	79 (2.78)
CCB4	12 (27.91)	19 (44.18)	1 (2.33)	11 (25.58)	43 (1.47)	28 (28.28)	46 (46.47)	1 (1.01)	24 (24.24)	99 (3.38)
CCB5	10 (21.28)	22 (46.81)	6 (12.77)	9 (19.14)	47 (1.42)	34 (36.96)	31 (33.70)	7 (7.61)	20 (21.73)	92 (2.78)
CCB6	41 (27.52)	56 (37.58)	11 (7.38)	41 (27.52)	149 (4.60)	44 (34.11)	35 (27.13)	10 (7.75)	40 (31.01)	129 (3.98)
CCB8	75 (12.25)	95 (15.52)	181 (29.58)	261 (42.65)	612 (18.21)	40 (10.15)	300 (76.14)	10 (2.54)	44 (11.17)	394 (11.73)

**Table 5:** The distributions of dysregulated genes in different gene expression levels.

Comparisons	Low (1 < TPM < 10)				Medium (10 < TPM < 100)				High (TPM > 100)			
	Up	Down	Total	Ratio	Up	Down	Total	Ratio	Up	Down	Total	Ratio
CCB1 vs. CC	309	439 **	748	5.69%	123	339 **	462	4.50%	13	62 **	75	5.60%
CCn1 vs. CC	144	176	320	2.43%	46	127 **	173	1.69%	5	15	20	1.49%
CCB4 vs. CC	140	268 **	408	3.10%	55	160 **	215	2.10%	6	15	21	1.57%
CCn4 vs. CC	47	64	111	0.84%	15	49 **	64	0.62%	1	3	4	0.30%
CCB5 vs. CC	122	180 **	302	2.30%	47	130 **	177	1.72%	4	22 **	26	1.94%
CCn5 vs. CC	20	88 **	138	1.05%	30	59	89	0.87%	1	10	11	0.82%
CCB6 vs. CC	469	628 **	1097	8.34%	142	395 **	537	5.23%	10	98 **	108	8.06%
CCn6 vs. CC	171	128	299	2.27%	73	91	164	1.60%	8	15	23	1.72%
CCB8 vs. CC	2245	2098	4343	33.04%	1046	2465 **	3511	34.21%	147	514 **	661	49.33%
CCn8 vs. CC	1171 **	890	2061	15.68%	732	1078 **	1810	17.64%	93	216 **	309	23.06%

** The significantly dominant dysregulated group determined by chi-square (*p* < 0.001).

**Table 6:** The expression distributions of DEGs in MAALs between non-MAALs and CC.

Comparisons	Down in MAALs				Up in MAALs			
	Up- (%)	Down- (%)	Unchanged- (%)	Total (%)	Up- (%)	Down- (%)	Unchanged- (%)	Total (%)
CCn1 vs. CC	3 (0.36)	269 (32.02)	568 (67.62)	840 (100)	284 (40.92)	4 (0.58)	406 (58.50)	694 (100)
CCn4 vs. CC	1 (0.22)	172 (38.83)	270 (60.95)	443 (100)	239 (63.06)	0 (0)	140 (36.94)	379 (100)
CCn5 vs. CC	2 (0.60)	196 (59.04)	134 (40.36)	332 (100)	237 (70.54)	0 (0)	99 (29.46)	336 (100)
CCn6 vs. CC	8 (0.71)	383 (34.17)	730 (65.12)	1121 (100)	478 (41.86)	5 (0.44)	659 (57.70)	1142 (100)
CCn8 vs. CC	158 (3.11)	1802 (35.49)	3117 (61.40)	5077 (100)	2048 (37.09)	78 (1.41)	3396 (61.50)	5522 (100)

## Data Availability

Transcriptome data were deposited in NCBI under accession number: PRJNA955364.

## Supplementary Materials

The following supporting information can be downloaded at: https://www.mdpi.com/article/10.3390/plants12102029/s1, Figure S1: Identification of target plants in progeny populations of MAALs; Table S1: Summary of MAALs, non-MAALs, CC and BB sequencing data aligned to the reference genome; Table S2: Uneven distributions of DEGs of *trans*-effects across all chromosomes in each of comparisons between MAAL and CC per chromosome; Table S3: Primer sequences for the DEGs used in qRT-PCR assays and relative changes in expression.
