# Supplementary material for: Transcriptional Interactions of Single B-Subgenome Chromosome with C-Subgenome in B. oleracea-nigra Additional Lines
Source: Plants (Basel). 2023 May 18;12(10):2029. doi: 10.3390/plants12102029 (PMC10220956; doi:10.3390/plants12102029)
Supplement: Supplementary file 1 [file plants-12-02029-s001.zip › plants-2370764-supplementary.pdf]

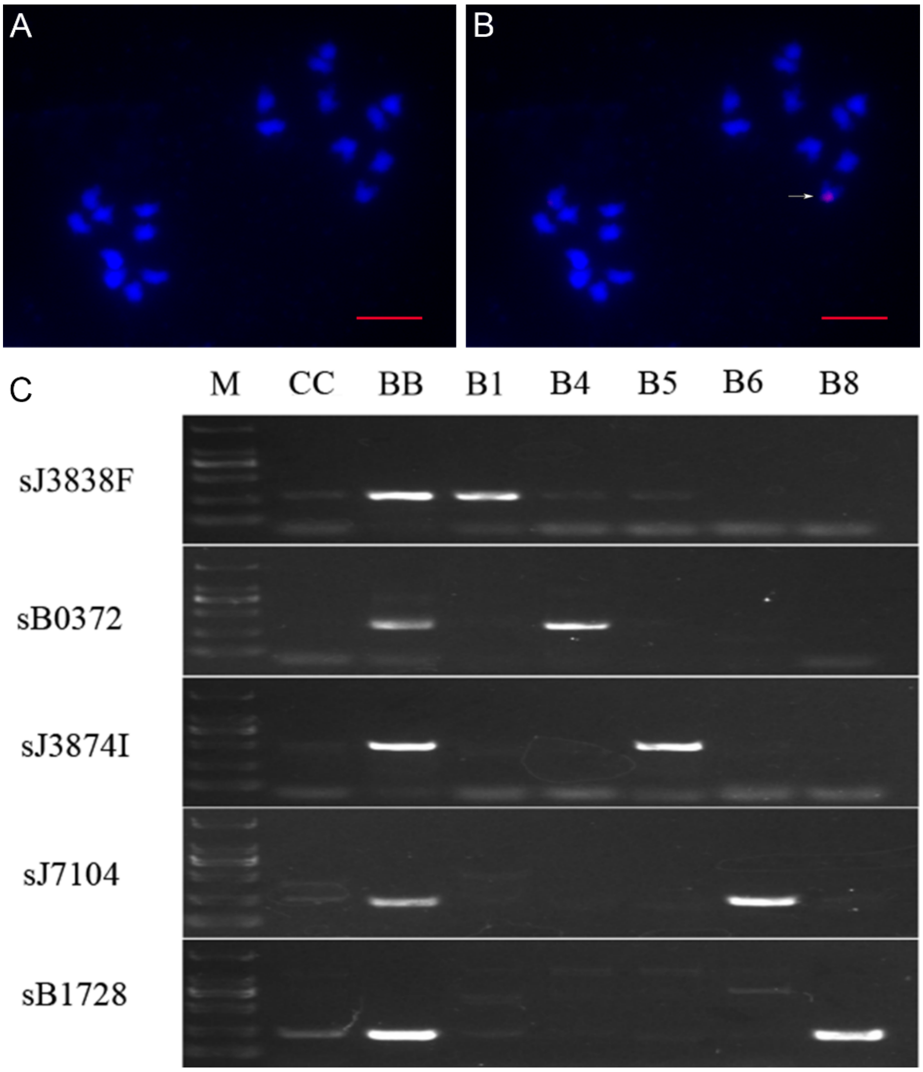

FIGURE S1 Identification of target plants in progeny populations of MAALs. (A−B) FISH using a B genome−specific probe demonstrates the target plant with the chromosome complement of 18C+1B. Blue indicates the DAPI counterstaining of chromosomes, and the red signal indicates the probe specifically for the B genome chromosome (B−cent, the PBNBH35 sub−sequence) (Bar, 5 μm). (C) SSR marker detection of plants with *B. nigra* chromosome. M, marker; CC, *B. oleracea*; BB, *B. nigra*; B1−B8, the plants with corresponding *B. nigra* chromosome respectively.

Table S1 Summary of MAALs, non−MAALs, CC and BB sequencing data aligned to the reference genome

Samples	Reads Number	Data Size	Q20	Q30	Alignment Rate	Reference genome
CCB1−1	38.82M	5.81Gb	0.987	0.954	92.84%	CC+B01
CCB1−2	40.52M	6.06Gb	0.9867	0.9529	92.29%	CC+B01
CCB1−3	40.31M	6.30Gb	0.9873	0.9543	92.40%	CC+B01
CCn1−1	44.35M	6.64Gb	0.9895	0.9613	93.41%	CC+B01
CCn1−2	39.91M	5.97Gb	0.9873	0.9549	93.08%	CC+B01
CCn1−3	39.63M	5.93Gb	0.987	0.9539	92.87%	CC+B01
CCB4−1	46.92M	7.02Gb	0.9882	0.9575	93.25%	CC+B04
CCB4−2	40.61M	6.08Gb	0.9882	0.955	93.51%	CC+B04
CCB4−3	40.94M	6.13Gb	0.9877	0.9556	93.46%	CC+B04
CCn4−1	37.33M	5.59Gb	0.9883	0.9567	93.29%	CC+B04
CCn4−2	43.17M	6.46Gb	0.988	0.9567	93.26%	CC+B04
CCn4−3	39.77M	5.95Gb	0.9882	0.9567	93.25%	CC+B04
CCB5−1	40.31M	6.03Gb	0.9868	0.9532	92.53%	CC+B05
CCB5−2	40.82M	6.11Gb	0.9873	0.9548	92.81%	CC+B05
CCB5−3	40.75M	6.10Gb	0.9878	0.9561	92.91%	CC+B05
CCn5−1	40.19M	6.01Gb	0.9864	0.9517	93.03%	CC+B05
CCn5−2	42.01M	6.29Gb	0.9875	0.9551	93.52%	CC+B05
CCn5−3	41.04M	6.14Gb	0.9874	0.9548	93.01%	CC+B05
CCB6−1	44.09M	6.60Gb	0.9873	0.9544	92.60%	CC+B06
CCB6−2	39.99M	5.98Gb	0.9875	0.955	92.56%	CC+B06
CCB6−3	40.20M	6.01Gb	0.9873	0.9546	92.69%	CC+B06
CCn6−1	39.59M	5.92Gb	0.9856	0.9491	93.12%	CC+B06
CCn6−2	44.97M	6.73Gb	0.9883	0.9579	93.30%	CC+B06
CCn6−3	44.03M	6.59Gb	0.9897	0.9623	92.96%	CC+B06
CCB8−1	37.83M	5.66Gb	0.9874	0.9525	88.66%	CC+B08
CCB8−2	42.64M	6.38Gb	0.9873	0.9524	88.99%	CC+B08
CCB8−3	43.78M	6.55Gb	0.9871	0.9516	89.31%	CC+B08
CCn8−1	42.46M	6.36Gb	0.9879	0.9548	92.84%	CC+B08
CCn8−2	37.81M	5.66Gb	0.9881	0.9549	91.53%	CC+B08
CCn8−3	41.29M	6.18Gb	0.9876	0.9532	89.94%	CC+B08
CC−1	43.13M	6.46Gb	0.9882	0.957	93.39%	CC
CC−2	40.34M	6.04Gb	0.9881	0.9569	93.61%	CC
CC−3	42.46M	6.35Gb	0.9878	0.9561	93.61%	CC
BB−1	40.65M	6.08Gb	0.9882	0.9568	82.01%	BB
BB−2	41.14M	6.16Gb	0.9879	0.9565	86.15%	BB
BB−3	42.75M	6.40Gb	0.9887	0.9588	87.52%	BB

Table S2 Uneven distributions of DEGs of *trans*−effects across all chromosomes in each of comparisons between MAAL and CC per chromosome

Chr	Total genes	CCB1 VS CC		CCB4 VS CC		CCB5 VS CC		CCB6 VS CC		CCB8 VS CC	
		DEGs	Ratio (%)	DEGs	Ratio (%)	DEGs	Ratio (%)	DEGs	Ratio (%)	DEGs	Ratio (%)
C1	5960	200	3.36^{a}	104	1.74^{a}	79	1.33^{abc}	262	4.40^{ab}	1080	18.12^{ab}
C2	6603	167	2.53^{bcd}	82	1.24^{bc}	50	0.76^{c}	204	3.09^{c}	1031	15.61^{c}
C3	9084	271	2.98^{ad}	150	1.65^{a}	146	1.61^{b}	376	4.14^{ab}	1679	18.48^{ab}
C4	7140	181	2.54^{bcd}	98	1.37^{abc}	60	0.84^{ac}	336	4.71^{b}	1205	16.88^{bc}
C5	6371	107	1.68^{e}	50	0.78^{d}	61	0.96^{ac}	220	3.45^{acd}	1124	17.64^{abc}
C6	5289	115	2.17^{ce}	76	1.44^{abc}	49	0.93^{ac}	190	6.59^{abcd}	924	17.47^{abc}
C7	6353	148	2.33^{bc}	85	1.34^{abc}	61	0.96^{ac}	199	3.13^{cd}	1120	17.63^{abc}
C8	6134	173	2.82^{abd}	96	1.57^{ac}	87	1.42^{ab}	256	4.17^{abd}	1173	19.12^{a}
C9	7276	172	2.36^{bc}	81	1.11^{b}	75	1.03^{abc}	220	3.02^{c}	1263	17.36^{abc}

^{a,b,c,d,e} Different groups were caculated by chi−square ($p<0.05$)

Table S3 Primer sequences for the DEGs used in qRT−PCR assays and relative changes in expression.

Gene ID	Forward Sequence	Reverse Sequence
BolC1t05740H	CCGTTGGATGGTTCGGTGA	TGGAGCATTTCCGGCTAAGAT
BniB01g000740.2N.1	TCTTGGCGATTCAGAGAGCG	CCGTTATCTCCGGTGGACTC
BniB04g026360.2N.1	GATCCATGGAAAGGAGAAGAATGG	CCTTAGGCCAAGGCTTCCT
BniB05g014860.2N.1	GAGGAGTTTAAGATTGAGCACAGC	GTTTCACCGGAGGGATCAGA
BniB06g045130.2N.1	CGGATGGCGAAATGGTGGATG	CCGTCTCTGTCTTCGTTGAGC
BniB08g013480.2N.1	TCCTCTTGGGTGAAGCATTTCG	GCTTGAAAGTGTAAGGCTGAGG
Actin	AGGTCTTGTTCCAGCCATCG	TGGTGCAAGTGCTGTGATCT
